# Systemic Consequences of Inflammatory Bowel Disease Beyond Immune-Mediated Manifestations

**DOI:** 10.3390/jcm14227984

**Published:** 2025-11-11

**Authors:** Antonio M. Caballero-Mateos, Eduard Brunet-Mas, Beatriz Gros

**Affiliations:** 1 Department of Internal Medicine, Gastroenterology Unit, Hospital Santa Ana, 18600 Motril, Spain; ogy1492@hotmail.com; 2 Institute of Biosanitary Research (IBS) Precision Medicine, 18012 Granada, Spain; 3 Gastroenterology Department, Parc Taulí University Hospital, Institut d’Investigació i Innovació Parc Taulí (I3PT-CERCA), Universitat Autònoma de Barcelona, 08208 Sabadell, Spain; 4 Centro de Investigación Biomédica en Red de Enfermedades Hepáticas y Digestivas (CIBERehd), Instituto de Salud Carlos III, 28029 Madrid, Spain; begrosal@gmail.com; 5 Department of Gastroenterology and Hepatology, Reina Sofía University Hospital, Maimonides Institute of Biomedical Research (IMIBIC), University of Cordoba, 14004 Cordoba, Spain

**Keywords:** inflammatory bowel disease, Crohn’s disease, ulcerative colitis, extraintestinal manifestations, immune-mediated diseases

## Abstract

Inflammatory bowel disease (IBD) management traditionally focuses on intestinal inflammation, yet extraintestinal manifestations can substantially impair patient quality of life. In this perspective, we emphasize the broad systemic impact of IBD—from highly prevalent conditions such as anemia, metabolic dysfunction-associated steatotic liver disease, or fatigue to rare but severe complications like interstitial lung disease and drug-induced glomerulonephritis. We review underlying mechanisms linking gut inflammation to distant organs, including immune dysregulation, microbial translocation, and metabolic derangements. Advances in diagnostics—such as biomarker panels, high-resolution imaging, and genomic/microbiome profiling—enable early detection and risk stratification. Emerging therapies, including targeted biologics (anti-TNF, anti-integrin, anti-IL-23), JAK and S1P modulators, precision nutrition, and microbiome modulation, offer new opportunities to address systemic inflammation. A multidisciplinary framework integrating gastroenterology with hepatology, hematology, neurology, nephrology, endocrinology, dermatology, pulmonology, and cardiology is essential to recognize hidden complications, facilitate timely intervention, and deliver personalized, comprehensive care for IBD.

## 1. Core Tip

This perspective aims to expand the clinical focus of IBD beyond the gut by highlighting its most common and its rare extraintestinal manifestations, advocating for multidisciplinary strategies and advanced diagnostics to improve patient outcomes.

## 2. Introduction

**Figure 1:**
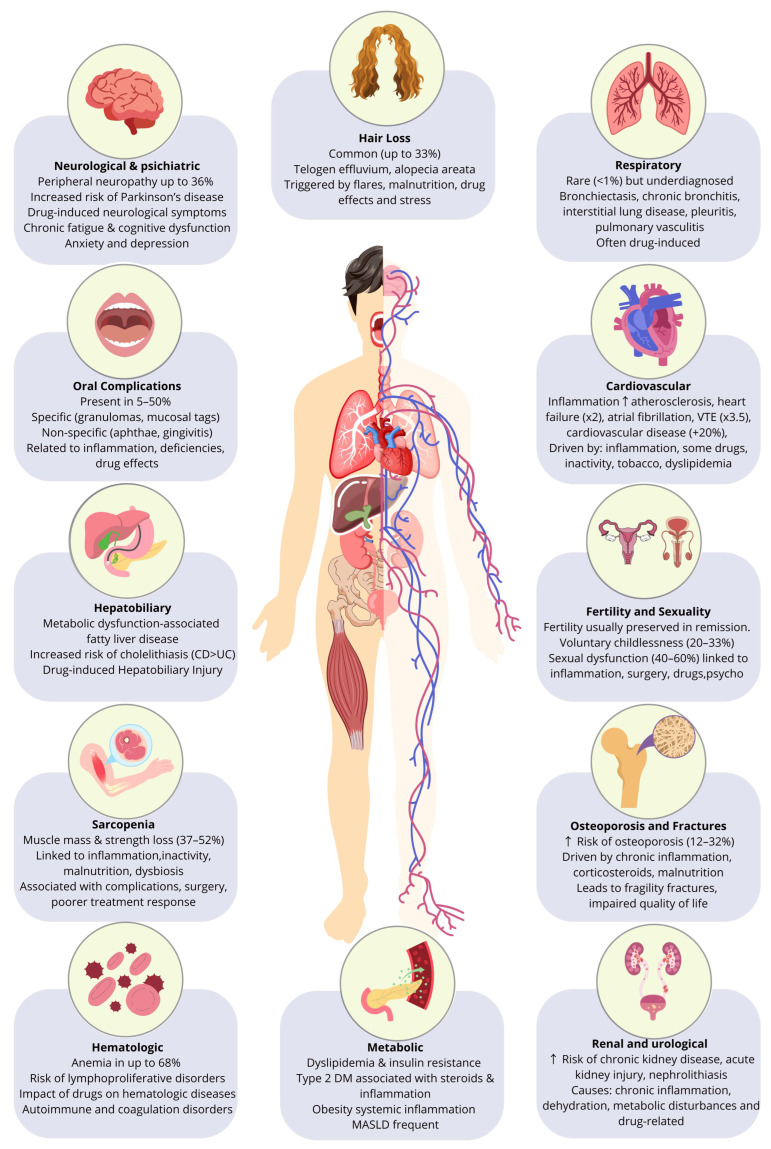
Systemic Consequences Of Inflammatory Bowel Disease Beyond Immune-Mediated Manifestations.

Inflammatory Bowel Disease (IBD), encompassing Crohn’s disease (CD) and ulcerative colitis (UC), primarily characterized by chronic, relapsing inflammation of the gastrointestinal tract, is in reality a systemic disease. Beyond its direct intestinal impact and well-recognized extraintestinal manifestations (ElMs) and immune-mediated inflammatory diseases (IMIDs), accumulating evidence highlights a broad spectrum of systemic consequences that arise as a direct result of chronic intestinal inflammation, therapies, surgeries or other. These consequences include metabolic disturbances, bone health alterations, nutritional deficiencies, growth impairment in pediatric populations, and increased risk of thromboembolic events, among others. Unlike ElMs and IMIDs, these systemic effects do not represent separate inflammatory conditions but rather reflect the prolonged inflammatory and metabolic burden of IBD itself. A schematic overview of the systemic consequences of IBD discussed below is shown in Figure 1.

Understanding these often-overlooked systemic repercussions is crucial for comprehensive patient care, as they can significantly affect quality of life, morbidity, and long-term prognosis. This review aims to summarize current knowledge on the systemic consequences of IBD, their underlying mechanisms, and their implications for clinical practice and research.

Reported prevalence rates vary widely across studies, reflecting the lack of meta-analyses and the methodological heterogeneity of available data, which mostly come from small or retrospective cohorts using different diagnostic criteria and definitions of disease activity.

## 3. Hepatobiliary Disorders

**Figure 2:**
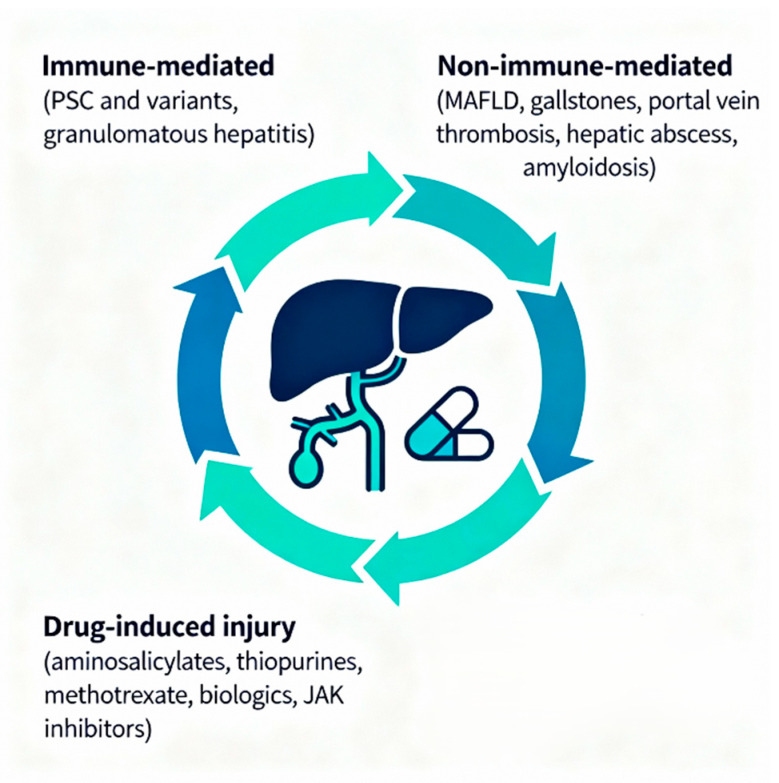
Hepatobiliary Disorders. Main pathogenic mechanisms.

Hepatobiliary manifestations represent some of the most clinically significant extraintestinal complications of IBD, affecting up to 30% of patients with both CD and UC [1,2]. These disorders can be broadly categorized into three main groups: immune-mediated conditions that typically follow an independent course from intestinal activity, non-immune-mediated disorders resulting from metabolic and physiological changes induced by IBD, and secondary conditions arising from therapeutic interventions (Figure 2).

- **Immune-Mediated Hepatobiliary Disorders:**

While immune-mediated hepatobiliary disorders such as primary sclerosing cholangitis (PSC), autoimmune hepatitis, granulomatous hepatitis and IgG4-related cholangitis represent important clinical complications in IBD, they fall outside the scope of this review as they constitute classical EIMs rather than systemic consequences of the chronic inflammatory and metabolic burden of IBD [3,4,5,6,7,8,9]. However, when present, these conditions impose significant systemic consequences requiring clinical attention: notably, PSC in patients with IBD dramatically increases colorectal cancer risk (4–6-fold higher than UC alone), necessitating annual colonoscopic surveillance from diagnosis [10]. Such multidisciplinary surveillance burden and metabolic alterations secondary to these hepatic disorders affect overall patient management and quality of life.

- **Non-Immune-Mediated Hepatobiliary Disorders**

Non-immune-mediated hepatobiliary manifestations in IBD arise from metabolic and physiological changes induced by the underlying intestinal disease process rather than from autoimmune mechanisms [11]. These conditions typically correlate with disease activity, nutritional status, and anatomical alterations resulting from IBD or its surgical management.

**Metabolic dysfunction-associated steatotic liver disease (MASLD)** represents an increasingly recognized complication in patients with IBD, with prevalence rates of 27–32% compared to 25% in the general population. The pathogenesis involves chronic systemic inflammation, gut microbiota dysbiosis, malnutrition, and medication effects, particularly corticosteroids. Risk factors include obesity, older age, hypertension, diabetes, and history of intestinal resection [2,12]. Corticosteroid exposure emerges as a particularly important modifiable risk factor through mechanisms involving hepatic lipogenesis and insulin resistance. Patients with IBD may develop MASLD even without traditional metabolic risk factors, and anti-TNF therapy may have protective effects.

**Cholelithiasis**: Gallstone disease shows significantly increased prevalence in CD patients (13–34%) versus UC patients who demonstrate similar rates to the general population. The risk is particularly elevated with ileal involvement or resection, where prevalence reaches 34% [1,3]. Pathogenesis relates to bile acid malabsorption at the terminal ileum, leading to bile acid pool depletion and cholesterol supersaturation. Risk factors include ileal disease location, previous resection, disease duration, and total parenteral nutrition use.

**Hepatic Abscess**: Pyogenic liver abscesses occur predominantly in CD (6.7 per 10,000 person-years), typically as multiple lesions from intra-abdominal sources. Risk factors include penetrating disease, abscesses, malnutrition, and corticosteroids [13].

- **Drug-Induced Hepatobiliary Injury**

Secondary hepatobiliary conditions in IBD predominantly arise from pharmacologic therapies and represent a spectrum of drug-induced liver injuries (DILI), ranging from asymptomatic enzyme elevations to acute liver failure. The EASL guidelines stipulate that, to diagnose DILI, all other potential causes of liver injury must be excluded and evidence of biochemical recovery following withdrawal of the suspected agent is required to establish causality [14].

Aminosalicylates, including mesalamine and sulfasalazine, are associated with rare hepatotoxicity, occurring in approximately 3–6 cases per million users, typically presenting as transient, mild transaminase elevations that resolve upon drug withdrawal; routine liver function monitoring is not recommended [15]. Thiopurines (azathioprine and 6-mercaptopurine) induce DILI in ~3% of patients with IBD (annual incidence 1.4%), mediated by both idiosyncratic reactions and dose-dependent hypermethylation to 6-methylmercaptopurine. Elevated 6-methylmercaptopurine levels correlate with hepatotoxicity [16]. Thiopurine S-methyltransferase activity predicts risk, and metabolite monitoring guides dose adjustments. Switching to allopurinol co-therapy with dose reduction mitigates toxicity in refractory cases. Methotrexate causes transaminase elevations in 10–11% of patients, with risk exacerbated by alcohol use, diabetes, and obesity. Folic acid supplementation reduces hepatotoxicity. Persistent elevations > 2× upper limit of normal after cumulative doses > 1.5 g warrant liver biopsy to assess fibrosis, though noninvasive fibrosis markers are under investigation [17].

Anti-TNF agents (infliximab, adalimumab) rarely cause mild ALT elevations (≈6%), with serious DILI in <1% of users. ALT elevations ≥ 3× upper limit require drug discontinuation; switching to an alternative anti-TNF or class may be safe [18]. Additionally, cases of drug-induced autoimmune-like hepatitis triggered by anti-TNF therapy have been reported. This condition mimics autoimmune hepatitis with characteristic interface hepatitis on liver biopsy and presence of autoantibodies. It is believed to result from disruption of immune tolerance due to altered TNF signaling, typically improving after drug withdrawal and immunosuppressive treatment [18]. Gut-selective anti-integrin therapy (vedolizumab) exhibits minimal hepatotoxicity, with isolated case reports of mild transaminase elevations [19]. Ustekinumab (anti-IL-12/23) is associated with mild ALT elevations (<1.4%), without significant DILI signals in clinical trials; newer p19-specific IL-23 inhibitors (e.g., risankizumab, mirikizumab) demonstrate similar hepatic safety profiles [20]. Tofacitinib (JAK inhibitor) data from rheumatoid arthritis reveal ALT elevations in 30% of patients, though significant DILI (>3× upper limit) occurs in only 1–2%; hepatotoxicity in IBD appears transient and reversible upon dose modification. JAK inhibitors beyond tofacitinib, including filgotinib and upadacitinib, demonstrate patterns of mild, transient ALT elevations in <2% of patients, with no cases of clinically significant liver injury requiring treatment discontinuation [21]. Sphingosine-1-phosphate modulators such as etrasimod exhibit excellent hepatic safety profiles in clinical trials [22].

Overall, DILI in patients with IBD necessitates baseline liver testing and periodic monitoring tailored to agent-specific risk, with prompt intervention—dose adjustment, drug cessation, or switch—to prevent progression to serious liver injury.

## 4. Neurological Disorders

Neurological complications represent significant but underrecognized extraintestinal manifestations of patients with IBD, occurring in 0.2–7.5% of patients according to different series. The recognition of neurological symptoms in patients with IBD is essential as they may significantly impact quality of life and require specific therapeutic considerations that may influence IBD treatment choices [23].

- **Peripheral Neuropathies**

**Peripheral neuropathies** represent the most frequent neurological complication in patients with IBD, affecting up to 36% of patients depending on diagnostic rigor. Most commonly, patients develop a sensorimotor polyneuropathy, characterized by symmetrical distal weakness, sensory loss, and diminished reflexes. Small-fiber neuropathy may present with burning pain and dysesthesia despite normal nerve conduction studies, requiring skin biopsy for definitive diagnosis [23].

**Guillain-Barré syndrome** (GBS) shows a well-documented association with IBD, particularly during disease flares or following gastrointestinal infections. The pathophysiology involves molecular mimicry between intestinal bacteria and peripheral nerve antigens, triggering cross-reactive autoimmune responses. Clinical presentation typically follows the classic ascending paralysis pattern [24]. Chronic inflammatory demyelinating polyneuropathy (CIDP) occurs with increased frequency in patients with IBD compared to the general population. Unlike GBS, CIDP follows a chronic progressive or relapsing-remitting course and may respond to immunosuppressive therapy used for IBD management.

The etiology of peripheral neuropathies in IBD is multifactorial [25]. Immune-mediated mechanisms involve shared HLA associations (particularly HLA-DR2 and HLA-DQ1) and cross-reactivity between gut antigens and peripheral nerve components. Nutritional deficiencies substantially contribute. Vitamin B12 deficiency, prevalent in CD with ileal involvement or resection, results in subacute combined degeneration. Folate deficiency from sulfasalazine or malabsorption exacerbates neuropathy risk, while thiamine deficiency in malnourished patients with IBD can cause Wernicke-like encephalopathy with peripheral neuropathy features, Table 1. Diagnosis relies on nerve conduction studies, quantitative sensory testing, and targeted laboratory assessment of vitamin levels, thyroid function, and autoantibodies [26].

- **Demyelinating Diseases**

Patients with IBD exhibit increased risk of central demyelinating disorders, including multiple sclerosis (MS) and isolated optic neuritis, with pooled hazard ratio of 1.54 for MS [27]. Shared HLA associations (DRB1*15:01) and Th17-mediated immunity suggest overlapping pathogenesis. Anti-TNF therapies can paradoxically induce demyelination in 0.05–0.2% of users, necessitating prompt drug discontinuation. In such cases, switching to vedolizumab, ustekinumab or anti-IL-23 is recommended to avoid exacerbating CNS lesions [27].

- **Posterior Reversible Encephalopathy Syndrome**

Rare cases of posterior reversible encephalopathy syndrome (PRES) have been reported in association with ustekinumab therapy. Although the causal link remains uncertain, clinicians should maintain a high index of suspicion in patients presenting with acute neurological symptoms such as seizures, visual disturbances, or altered mental status. Prompt recognition and drug withdrawal typically result in complete resolution of symptoms [28].

- **Neurodegeneration and Gut–Brain Axis**

Emerging evidence suggests increased risk of neurodegenerative diseases in patients with IBD, highlighting the importance of the gut–brain axis in neurodegeneration. Recent meta-analyses demonstrate increased risk of Parkinson’s disease (pooled HR 1.39, 95% CI 1.05–1.85) and dementia (pooled HR 1.22, 95% CI 1.02–1.46) in patients with IBD [29]. Pathophysiologic mechanisms involve chronic systemic inflammation leading to neuroinflammation, disruption of the blood–brain barrier, alpha-synuclein aggregation initiated in the enteric nervous system, vitamin D deficiency and dysbiosis of the gut microbiota affecting neurotransmitter production and short-chain fatty acid metabolism. Serum biomarkers linking IBD to neurodegeneration include elevated neurofilament light chain (NfL), neuron-specific enolase (NSE), and inflammatory cytokines such as IL-6 and TNF-α. Interestingly, some IBD treatments may provide neuroprotective effects. Anti-TNF therapy has been associated with reduced Parkinson’s disease risk in some cohorts, possibly through reduction of systemic inflammation [27]. Similarly, aminosalicylates may have protective effects against cognitive decline through anti-inflammatory mechanisms [30].

- **Drug-Induced Neurological Complications**

As previously noted, anti-TNF agents may induce demyelinating complications and warrant the precautions described earlier. Metronidazole-induced peripheral neuropathy occurs in a dose- and duration-dependent manner, typically presenting as distal sensory symptoms with burning pain and numbness. The mechanism involves mitochondrial dysfunction and oxidative damage to peripheral nerves. Symptoms usually improve with drug discontinuation but may persist for months. Other agents associated with neurological complications include ciclosporin (posterior reversible encephalopathy syndrome, tremor), corticosteroids (steroid myopathy, psychiatric symptoms), and methotrexate (rare leukoencephalopathy) [24]. Management strategies involve prompt recognition of symptoms, drug discontinuation when appropriate, and consideration of alternative therapeutic approaches, Table 1.

## 5. Psychological and Psychiatric Disorders

Psychological and psychiatric comorbidities are prevalent in patients with IBD, affecting approximately 30–40% of patients and exerting a profound impact on treatment adherence, disease activity, and overall quality of life [31]. These disorders can be broadly grouped into chronic fatigue with cognitive dysfunction, mood and anxiety disorders, and social–occupational limitations.

- **Chronic Fatigue and Cognitive Dysfunction**

**Chronic fatigue** is characterized by persistent, debilitating exhaustion that is disproportionate to exertion and not alleviated by rest. Up to 80% of patients report clinically significant fatigue, even during periods of remission. The pathophysiology is multifactorial [31]:

o  Systemic inflammation: Elevated circulating IL-6 and TNF-α cross the blood–brain barrier and activate microglia, inducing central fatigue through neuroimmune mechanisms.
o  Anemia and nutritional deficiencies, such as iron or vitamin B12 depletion, should be identified and treated promptly, as they significantly contribute to fatigue in IBD
o  Sleep disturbance and pain: Nocturnal symptoms and abdominal pain fragment sleep architecture, further exacerbating fatigue.

Assessment requires both subjective and objective measures: the IBD-Fatigue Scale and the FACIT-F questionnaire quantify symptom severity; laboratory evaluation includes complete blood count, iron studies, B12 levels, and inflammatory markers, Table 1. Management strategies include treating underlying inflammation aggressively to reduce cytokine burden. Correct anemia and nutritional deficiencies. Structured exercise programs show promise, with moderate aerobic and resistance training improving quality of life and potentially reducing inflammatory markers [30]. Cognitive-behavioral therapy (CBT) specifically adapted for IBD fatigue has demonstrated efficacy in reducing subjective fatigue scores, with patients preferring CBT over other psychological interventions [31]. Mindfulness-based cognitive therapy (MBCT) has shown significant reductions in subjective fatigue experience (Cohen’s d = 0.46), with 36% achieving clinically relevant improvement versus 10% in controls [32].

**Cognitive dysfunction**, often termed “brain fog,” affects approximately 30% of patients with IBD and presents with impairments in memory, attention, and executive function. Risk factors include active flares, long-term corticosteroid exposure, and coexistent depression [33]. Mechanistic contributors mirror those of fatigue: cytokine-mediated neuroinflammation, dysbiosis of the gut microbiota, and increased intestinal permeability facilitate translocation of microbial metabolites that disrupt neuronal function. Neuropsychological evaluation using the Montreal Cognitive Assessment (MoCA) and Trail Making Test, complemented by patient-reported outcome measures, guides the diagnosis. Interventions focus on mood optimization, cognitive rehabilitation exercises, and investigational use of psychobiotics to modulate the gut–brain axis [34].

- **Mood Disorders and Anxiety**

**Depressive and anxiety disorders** are highly comorbid with patients with IBD, with lifetime depression rates of 20–25% and anxiety rates of 30–40%. A bidirectional relationship exists: depression and anxiety exacerbate IBD activity through stress-mediated activation of the hypothalamic–pituitary–adrenal axis and proinflammatory pathways, while active intestinal inflammation worsens mood and anxiety symptoms [35]. These psychiatric conditions serve as important mediators in the association between IBD and suicidality. Recent systematic reviews demonstrate that patients with IBD have a 17.3% prevalence of suicidal ideation, with significantly increased risk of suicide attempts (RR 1.39) and completed suicide (RR 1.25) compared to controls, particularly among patients with CD, female patients, and those with pediatric-onset disease [36].

Screening tools such as the Patient Health Questionnaire-9 (PHQ-9) and the Generalized Anxiety Disorder-7 (GAD-7) should be implemented routinely in IBD clinics to ensure early detection, Table 1. Treatment modalities include: Pharmacotherapy: Selective serotonin reuptake inhibitors (e.g., sertraline, escitalopram) demonstrate efficacy in reducing depressive and anxiety symptoms and may indirectly modulate immune function. Psychotherapy: Cognitive-behavioral therapy (CBT) reduces maladaptive coping behaviors, improves resilience, and has been shown to enhance quality of life and reduce relapse rates [35]. Stress-reduction techniques: Mindfulness-based stress reduction and relaxation training attenuate HPA axis hyperactivity and improve psychological well-being [37].

- **Social Isolation and Occupational Limitations**

Social and occupational impairments are common yet underappreciated in patients with IBD. Stigmatization related to urgency, incontinence, and frequent health care utilization leads approximately 50% of patients to self-isolate, resulting in loneliness, decreased social capital, and heightened depression risk [38]. Occupational dysfunction involves both absenteeism and presenteeism. Patients with IBD lose an average of 10–20 workdays per year to flares, medical appointments, and fatigue, while presenteeism further reduces productivity. The economic burden extends beyond direct health care costs to include lost wages and decreased workplace performance [38].

Evidence-based interventions include workplace accommodations, occupational therapy assessments, which guide ergonomic adjustments and energy-conservation strategies to support sustained employment, and peer-support and counseling [39].

An integrated, multidisciplinary care model, incorporating gastroenterology, psychiatry, psychology, nutrition, and social work, is essential to address the complex interplay between IBD and psychological disorders. Routine mental health screening protocols and fatigue assessments should be embedded within IBD clinics.

## 6. Hematologic Disorders

Patients with IBD develop diverse hematologic complications. While thrombotic complications will be discussed in following sections, this one addresses anemia, lymphoproliferative disorders, and other blood abnormalities. Regular hematologic monitoring requires complete blood counts, particularly during immunosuppressive therapy. Lymphoma risk stratification includes age, male sex, thiopurine duration, and combination immunosuppression. For cytopenias, drug-related causes require exclusion before attributing to IBD activity. The benefit-risk ratio generally favors continued immunosuppression despite hematologic risks, though individual assessment considering age, comorbidities, and treatment history guides optimal decisions.

- **Anaemia:**

Anaemia affects up to 68% of patients with IBD and significantly impairs quality of life. Iron deficiency anaemia (IDA) and anaemia of chronic disease (ACD) are the most frequent types, either as sole conditions or in conjunction. IDA results from chronic blood loss, malabsorption, and dietary restrictions, while ACD occurs through hepcidin-mediated iron sequestration and cytokine-suppressed erythropoiesis [40].

Diagnostic workup should follow a systematic approach starting with evaluation of mean corpuscular volume (MCV), though a normal MCV does not exclude iron deficiency, as up to 40% of pure IDA cases are normocytic. The minimum diagnostic panel should include complete blood count with MCV, reticulocyte count, serum ferritin, transferrin saturation (TSAT), and C-reactive protein (CRP). In the presence of inflammation, diagnostic interpretation becomes challenging, as ferritin is an acute-phase reactant. Under these circumstances, serum ferritin > 100 µg/L with TSAT < 20% indicates ACD, while ferritin between 30–100 µg/L with TSAT < 16% suggests a combination of IDA and ACD [41]. Novel indices such as soluble transferrin receptor-to-log ferritin ratio and reticulocyte haemoglobin content (CHr < 29 pg) may provide additional diagnostic accuracy when initial tests are inconclusive [42].

Treatment aims to normalize both haemoglobin levels and iron stores, as correction improves quality of life independently of clinical activity. Intravenous iron is recommended as first-line therapy in patients with clinically active IBD, severe anaemia (Hb < 10 g/dL), previous intolerance to oral iron, or when erythropoiesis-stimulating agents are required, demonstrating superior efficacy and faster response compared to oral preparations. Multiple formulations are available, including iron sucrose, ferric carboxymaltose, and ferric derisomaltose, with similar efficacy profiles (Table 1). Oral iron may be considered in patients with mild anaemia (Hb > 11 g/dL) whose disease is clinically inactive, using alternate-day dosing (60–120 mg ferrous salt) to optimize absorption and minimize gastrointestinal side effects. Erythropoiesis-stimulating agents combined with intravenous iron may benefit patients with ACD refractory to iron therapy alone, maintaining target haemoglobin ≤ 12 g/dL to minimize thromboembolic risk. Following treatment, regular monitoring every 3–6 months is essential, as anaemia recurs rapidly in up to 50% of patients within 10 months, with addressing underlying inflammation remaining paramount [42].

- **Lymphoproliferative Disorders:**

Patients with IBD face increased lymphoma risk, particularly with immunosuppressive therapy. Meta-analyses show untreated patients have similar lymphoma rates to the general population, while thiopurine recipients demonstrate 4-fold increased risk (0.02–0.16 per 1000 patient-years) [41]. Non-Hodgkin lymphoma predominates, with rare but aggressive hepatosplenic T-cell lymphoma affecting young males on thiopurines. Risk correlates with immunosuppression duration and intensity; combination therapy (thiopurines + anti-TNF) confers higher risk than monotherapy. Anti-TNF monotherapy shows conflicting lymphoma data, while newer biologics lack sufficient long-term safety data [43].

- **Quantitative Hematologic Abnormalities:**

Thrombocytosis occurs in up to 78% of patients, correlating with disease activity through IL-6 and thrombopoietin upregulation. While generally benign, severe thrombocytosis (>1,000,000/μL) may paradoxically increase bleeding risk via acquired von Willebrand disease [44]. Leukopenia and neutropenia affect 5–15% of patients, primarily from drug-induced bone marrow suppression by thiopurines, methotrexate, or sulfasalazine, Table 1. Thiopurine-induced leukopenia results from TPMT deficiency or dose-dependent toxicity. Severe neutropenia (<500/μL) requires immediate drug cessation and potential G-CSF support [45].

- **Autoimmune and Coagulation Disorders:**

Autoimmune hemolytic anemia occurs rarely, typically with Coombs-positive warm antibodies reflecting shared autoimmune pathways. Immune thrombocytopenic purpura may develop independently, requiring balanced management of IBD immunosuppression and ITP treatment [42]. Beyond hypercoagulability discussed previously, patients with IBD may develop factor XIII deficiency during active inflammation and vitamin K deficiency from malabsorption, leading to delayed bleeding and prolonged coagulation times [44].

## 7. Renal and Urological Disorders

IBD is associated with increased risks of chronic kidney disease (CKD), acute kidney injury (AKI), nephrolithiasis, and specific glomerular and tubulointerstitial diseases, warranting regular assessment of renal function and vigilance for urological complications in this population. The pathogenesis is multifactorial, involving both disease-related and treatment-related mechanisms.

CKD and AKI in patients with IBD are linked to chronic systemic inflammation, immune dysregulation, and metabolic disturbances [46]. Persistent inflammation can promote renal injury through cytokine-mediated pathways and oxidative stress, while dehydration from diarrhea or malabsorption increases susceptibility to AKI [47]. Surgical resections, especially in CD, further elevate CKD risk, likely due to altered fluid and electrolyte handling and increased risk of nephrolithiasis [3,4]. Pediatric and adult cohorts both demonstrate a measurable decline in estimated glomerular filtration rate (eGFR) over time, supporting the need for routine renal monitoring in patients with IBD [5,6].

Nephrolithiasis is more common in patients with IBD, particularly CD, due to enteric hyperoxaluria from fat malabsorption, chronic diarrhea, and altered urinary pH [48]. These factors increase urinary oxalate and decrease citrate, predisposing to calcium oxalate stone formation [49].

Epidemiological data indicate that patients with IBD have a significantly increased risk of CKD (OR 1.59, 95% CI 1.31–1.93) [50], AKI (HR 1.97, 95% CI 1.70–2.29), and nephrolithiasis (HR 1.69, 95% CI 1.48–1.93) [51,52] compared to the general population, with 10-year absolute risks for CKD and kidney stones of 11.8% and 5.6%, respectively [53,54]. These risks are similar for CD and UC, and are independent of genetic predisposition to kidney disease [55,56]. Renal manifestations are more common in patients with active disease, prior surgery, or severe IBD [57,58].

On the other hand, tubulointerstitial nephritis arises from both direct extraintestinal immune-mediated injury and drug-induced nephrotoxicity, especially from aminosalicylates. Kidney damage induced by medications in patients with IBD most commonly involves aminosalicylates (mesalazine/mesalamine), calcineurin inhibitors, methotrexate, and, less frequently, biologics, Table 1 and Table 2. Aminosalicylates are the best-established cause, with acute or chronic tubulointerstitial nephritis (TIN) as the primary lesion [59]. TIN can present with non-specific symptoms (fatigue, malaise, low-grade fever), and laboratory findings may include rising serum creatinine, sterile pyuria, and mild proteinuria. Renal biopsy typically shows interstitial inflammation, sometimes with granulomas or eosinophils, and discontinuation of the offending drug is the mainstay of management [13,14]. Biologics (anti-TNF, anti-integrin, anti-IL-12/23) are rarely implicated in nephrotoxicity [8,13,15]. JAK inhibitors (e.g., tofacitinib) may require dose reduction in advanced CKD, but direct nephrotoxicity is not well established [60]. However, cases in drug-naïve patients suggest a primary IBD-related immune mechanism as well [8,14].

Glomerulonephritis, especially IgA nephropathy, is overrepresented in patients with IBD and is thought to result from aberrant mucosal immune responses in the gut leading to increased production and deposition of IgA-containing immune complexes in the glomeruli. This gut-kidney axis dysfunction is supported by the higher prevalence of IgA nephropathy in patients with IBD compared to the general population [61].

Risk factors for drug-induced renal complications in patients with IBD also include pre-existing CKD, older age, concomitant nephrotoxic medications (e.g., NSAIDs) and dehydration, Table 1.

## 8. Osteoporosis and Fractures

IBD is widely recognized as a significant risk factor for metabolic bone disease, including osteoporosis and low bone mineral density (BMD). The overall pooled prevalence of osteoporosis in IBD is reported to be 12.2% (95% CI: 9.1–15.3), although reported rates vary across different countries [62]. This represents a clinically meaningful increase compared to control populations, with IBD exhibiting a pooled odds ratio of 1.64 (95% CI: 1.24–2.16) for osteoporosis [62]. Differences in diagnostic criteria, characteristics of patient populations (such as age, disease severity, and duration), geographical locations, and the specific assessment methodologies employed poses a considerable challenge in establishing a precise global prevalence for osteoporosis in patients with IBD.

Bone loss in IBD is a multifactorial process, resulting from a complex interplay of chronic inflammation, prolonged corticosteroid use, and various nutritional deficiencies [63].

Chronic inflammation, intrinsic to IBD, directly impacts bone metabolism. Proinflammatory cytokines play a pivotal role in accelerating bone loss by influencing both bone resorption and formation [64,65].

A critical concern in IBD is the significantly increased risk of osteoporotic fractures. Some studies indicate a 32% increased risk compared to the general population [66]. Such fractures contribute substantially to morbidity, mortality, and a diminished quality of life.

Corticosteroids are well-established contributors to low BMD, osteoporosis, and an increased risk of fractures [67].

Malnutrition and specific nutritional deficiencies are also important contributors [28,68]. Additionally, low body mass is recognized as a factor contributing to bone disease in this population. Beyond these IBD-specific factors, general risk factors for osteoporosis, such as older age, female gender, smoking, and a family history of fracture, also contribute to bone loss in patients with IBD. Both the American Gastroenterological Association (AGA) [69] and the British Society of Gastroenterology (BSG) [70]) recommend calcium and vitamin D supplementation in IBD if at risk for or diagnosed with osteoporosis, additional therapies may be considered depending on age and DEXA results, Table 1.

BMD is primarily assessed using Dual-energy X-ray Absorptiometry (DEXA), reported as a T-score (for postmenopausal women and men ≥ 50 years) or a Z-score (for premenopausal women, younger adults, and children).

The International Society for Clinical Densitometry (ISCD) provides specific indications for BMD testing [71]. Men aged ≥70 years old, women aged ≥65 years old; Women during menopausal transition; Post-menopausal women < 65 years old, and men < 70 years old who possess specific risk factors; Adults with a history of fragility fracture, or a disease or pathological condition known to cause bone loss, such as IBD; Adults being considered for or currently taking high-risk medications known to cause low bone mass or bone loss (e.g., corticosteroids, JAK inhibitors) [72].

## 9. Sarcopenia

Sarcopenia, defined as a progressive loss of skeletal muscle mass and function, is a prevalent and serious systemic consequence of IBD that significantly impacts patient outcomes and overall quality of life [73]. Sarcopenia is highly prevalent in patients with IBD, a meta-analysis reported prevalence rates of 52% in CD and 37% in UC [74].

The development of sarcopenia in IBD is multifactorial and closely linked to the “gut–muscle axis,” shaped by chronic inflammation, gut microbiota alterations, malnutrition, and reduced physical activity [75,76]. Persistent inflammation in IBD elevates cytokines impair intestinal barrier function promote muscle cell apoptosis and protein breakdown.

Malnutrition, stemming from reduced intake, malabsorption, protein loss, and elevated energy demands, compounds this process. Deficiencies in vitamin D, zinc, and insulin-like growth factor-1 (IGF-1) are common and accelerate protein catabolism [77,78].

Lastly, physical inactivity, reported in nearly half of patients with IBD, often due to fear of exacerbations and lack of exercise guidance [79,80], further aggravates sarcopenia and related comorbidities.

Sarcopenia is consistently associated with a more severe disease course and adverse clinical outcomes in IBD [36,37]. Its impact is particularly notable in surgical contexts, where it significantly increases the likelihood of requiring surgery [81,82] and the risk of postoperative complications, adjusted data consistently identify sarcopenia as an independent predictor for both increased surgery rates (OR 2.65; 95% CI 1.12–6.33; *p* = 0.027) and postoperative complications (OR 6.09; 95% CI 1.75–21.17; *p* = 0.004) [83].

Beyond surgical outcomes, sarcopenia also influences treatment response [84,85]. It serves as an adverse prognostic factor for achieving endoscopic remission and is associated with primary loss of response to biological therapies, particularly anti-TNF agents [73]. In CD specifically, sarcopenia is associated with a higher risk of hospitalization (OR, 1.87, 95% CI 1.19–2.23) and the development of abscesses (OR, 5.03, 95% 2.05–12.38) [86]. This indicates that sarcopenia is not merely a consequence of severe disease but functions as an independent driver of morbidity. Therefore, active screening and management of sarcopenia should be integrated into routine IBD care, irrespective of overt disease activity.

Diagnostic approaches have evolved from focusing solely on muscle mass to incorporating muscle strength and physical performance, recognizing the functional consequences of muscle loss. Gold-standard imaging techniques like CT and MRI are limited in clinical practice due to cost, time, and radiation exposure, Table 1. More accessible methods, such as ultrasonography, are emerging as practical alternatives for early detection and intervention. However, the lack of IBD-specific functional cut-offs (such as handgrip strength, gait speed, and timed up and go tests) highlights the need for further refinement in diagnostic criteria [87].

Despite advancements in understanding and managing sarcopenia in IBD, several areas require further investigation of such sarcopenic obesity [78]. There is a need for more research to fully elucidate specific mechanisms contributing to sarcopenia, optimize therapeutic strategies, and evaluate the long-term efficacy of interventions across diverse patient populations.

## 10. Dermatological Manifestations: Hair Loss and Oral Complications in IBD

Hair loss is a frequent and distressing issue in patients with IBD, reported in approximately 33% of cases [88]. It usually presents as telogen effluvium, the most common form characterized by diffuse hair shedding triggered by IBD flares, protein–calorie malnutrition, and deficiencies in essential nutrients such as vitamin B12 and iron [89]. In some cases, hair loss can even precede the diagnosis of IBD, acting as an early clinical indicator. Alopecia areata, a patchy autoimmune-related hair loss, is also observed in these patients and shares genetic susceptibility factors with IBD [90]. A less common but more severe presentation is primary cicatricial alopecia, which leads to permanent follicular damage.

The underlying mechanisms of hair loss in IBD are multifactorial. Chronic inflammation and active disease states frequently coincide with telogen effluvium, which tends to improve when remission is achieved and recur during relapses, highlighting the influence of inflammatory activity. Nutritional deficiencies due to malabsorption, reduced dietary intake, and increased metabolic demands further exacerbate hair loss, particularly through deficiencies in iron, vitamin B12, and protein [89]. Medications commonly used to treat IBD can also play a role. Azathioprine has been reported to both induce hair loss and paradoxically promote hair regrowth in cases of alopecia universalis linked to UC [91]. Methotrexate and 6-mercaptopurine are additional immunosuppressants associated with hair loss [92]. The relationship between anti-TNF agents such and hair loss is complex, with reports describing paradoxical psoriasiform eruptions and alopecia areata-like lesions [93]. However, larger studies have demonstrated that anti-TNF therapy is actually associated with a lower risk of hair loss (OR = 0.28, 95% CI: 0.08–0.98), suggesting a possible protective effect [88]. Similarly, mesalamine has been linked to reduced odds of alopecia (OR = 0.43, 95% CI: 0.19–0.86) [88]. These contradictory findings suggest that drug-induced hair loss may be a rare adverse event or influenced by confounding factors, and overall, the control of IBD activity achieved with these therapies might help reduce hair loss rather than exacerbate it.

Management typically focuses on controlling intestinal inflammation, as hair shedding often resolves with disease remission. Addressing nutritional deficiencies, particularly iron and vitamin B12, is an essential part of treatment, Table 1. When a medication is suspected to be contributing to alopecia, therapeutic alternatives may be considered, although drug discontinuation is not always required. In some cases, topical treatments can improve hair regrowth without interrupting biologic therapy, although pediatric cases of TNF-alpha inhibitor-induced alopecia have shown better recovery after stopping the drug [94]. These observations highlight the need for individualized assessment and tailored management strategies in IBD-related hair loss.

Oral complications are common in IBD, affecting 5–50% of patients and often impairing quality of life [95]. These manifestations can be classified as specific or non-specific. Specific oral lesions, mainly observed in CD with a reported prevalence of 20–50%, are characterized by non-caseating granulomas and may precede gastrointestinal symptoms, aiding early diagnosis [96]. Clinically, they can present as cobblestoning of the oral mucosa, deep linear ulcerations, mucosal tags, mucogingivitis, and firm swelling of the lips and face [97]. Non-specific lesions occur in both CD and UC and are associated with chronic inflammation, nutritional deficiencies, and side effects of IBD medications [95]. These include aphthous stomatitis, angular cheilitis, pyostomatitis vegetans (more frequent in UC), gingivitis, periodontitis, glossitis, halitosis, dental erosion and caries [95]. The underlying mechanisms involve systemic inflammation and malnutrition, with iron, vitamin B12, folate, zinc, and vitamin D deficiencies frequently manifesting in the oral cavity [95]. Medications such as aminosalicylates, corticosteroids, thiopurines, methotrexate, calcineurin inhibitors, biologic therapies, and antibiotics can induce oral changes through direct toxicity, immunosuppression leading to opportunistic infections, or bone marrow suppression. Oral lesions hold diagnostic value, particularly in CD, where granulomatous inflammation can be histologically confirmed [96]. Management primarily focuses on controlling intestinal inflammation, as oral manifestations often improve with effective IBD treatment [98]. Multidisciplinary care involving gastroenterologists and oral medicine specialists is essential, Table 1. Topical agents, nutritional supplementation, antifungals, antivirals, or drug adjustments may be required for specific cases. Despite their clinical relevance, there is a lack of consensus guidelines for managing these manifestations, highlighting the need for further research into their mechanisms, the role of the oral microbiome, and standardized treatment recommendations.

## 11. Fertility and Sexuality

Fertility in patients with IBD is a complex issue influenced by biological, psychological, and treatment-related factors. In individuals with quiescent disease, overall fertility rates are generally similar to those of the general population. However, voluntary childlessness remains common, largely driven by concerns regarding pregnancy, heredity, and disease flare-ups [99,100]. This is closely associated with limited understanding of pregnancy-related issues in IBD and reflects a substantial psychological and emotional burden that extends beyond physiological infertility [101]. These findings underscore the importance of preconception counseling addressing both medical and psychosocial aspects.

Multiple factors influence fertility outcomes in IBD. Disease activity plays a critical role, as active inflammation can impair reproductive and sexual function in both men and women [57,58]. In men, oxidative stress associated with systemic inflammation negatively impacts sperm quality. In Crohn’s disease, pelvic involvement or prior surgery may reduce fertility through adhesions or structural complications [102]. Surgical interventions, particularly pelvic procedures such as proctocolectomy with ileal pouch-anal anastomosis (IPAA) or ileostomy, are well-recognized contributors to reduced fertility in women, primarily due to postoperative adhesions. For men, IPAA can cause retrograde ejaculation and erectile dysfunction, although sexual function often improves after surgery [103,104].

Medications can further influence fertility. Sulfasalazine can temporarily impair male fertility; discontinuation 3–4 months before conception is recommended. Methotrexate is teratogenic and contraindicated in both men and women planning conception [105]. In contrast, most other IBD treatments, including biologic agents, are considered safe during conception and pregnancy when risks and benefits are carefully discussed. Regarding newer treatment agents such as JAK inhibitors and S1P, for now the recommendation is to avoid during pregnancy and lactation.

Preconception counseling plays a central role in managing reproductive health in patients with IBD. Optimizing nutrition and achieving disease remission are key steps for individuals planning to conceive. Couples experiencing infertility after six months of unprotected intercourse should be referred to fertility specialists to explore reversible causes and alternative conception methods. In vitro fertilization is a viable option for women with IBD.

Sexual dysfunction is another common yet underrecognized issue in IBD, affecting 40–60% of women and 40–50% of men [106,107,108]. Disease activity strongly correlates with sexual dysfunction: men with active IBD report lower desire and satisfaction, while women often experience lubrication difficulties and pain during intercourse [103]. Perianal disease markedly increases the risk of sexual dysfunction, and vulvovaginal CD can profoundly disrupt intimate relationships [105].

Treatment-related factors also contribute to sexual problems. Long-term corticosteroid use can reduce testosterone levels, leading to diminished libido and erectile dysfunction [109]. Antidepressants, particularly SSRIs commonly used to manage depression in IBD, are associated with sexual side effects, whereas alternatives like SNRIs, mirtazapine, or bupropion may have fewer adverse effects [110]. Biologic therapies have not been conclusively linked to significant sexual dysfunction. Psychological factors also play major roles in sexual health impairment [103].

Managing sexual dysfunction in IBD requires a comprehensive and individualized approach. Controlling underlying disease activity is fundamental, as symptom improvement often correlates with restored sexual function. Optimizing nutrition, eliminating alcohol and tobacco use, treating depression, and reviewing medications that may contribute to sexual problems are essential steps. Personalized care plans tailored to specific concerns can significantly improve sexual health outcomes.

## 12. Cardiovascular and Thromboembolic Complications

Chronic inflammation is a well-established risk factor for atherosclerosis and vascular events. It promotes endothelial damage, oxidative stress, lipid accumulation, platelet adhesion, and plaque rupture [111,112]. In this sense, the systemic inflammatory state in patients with IBD (inflammatory interleukins, C-reactive protein, calprotectin, flare ups, use of steroids) can contribute to this process [113].

A meta-analysis by Singh et al. reported a 20% higher risk of cardiovascular disease in individuals with IBD [114]. This association has been observed in several real-world cohorts. A 2018 study from the Mayo Clinic found that patients with IBD had twice the risk of heart failure compared to controls, especially women with UC (HR 2.03; 95% CI, 1.36–3.03) [115]. Similarly, a Danish study showed a 37% increased risk of heart failure in patients with IBD [116]. Moreover, some studies suggest that IBD is associated with premature (age < 55) and even extremely premature (<40) atherosclerotic cardiovascular disease, particularly in younger individuals [117].

Although IBD therapies aim to reduce inflammation, some may influence in cardiovascular risk. Corticosteroids have been linked to heart failure and thromboembolic events [118], and high doses of salicylates have been associated with increased arterial stiffness and cardiovascular disease risk [119]. JAK inhibitors can raise lipid levels and were associated with more major cardiovascular events compared to TNF inhibitors [120].

Given these risks, prevention strategies are essential. While there are no specific cardiovascular prevention guidelines for IBD the European Society of Cardiology recognizes IBD as a cardiovascular risk factor, supporting a proactive approach similar to that used in other high-risk populations; suggest using coronary artery calcium scoring to guide statin use in intermediate-risk patients (>7.5% to <20%) [121,122]. Lifestyle measures—such as physical activity, weight control, and smoking cessation—are especially important to reduce cardiovascular risk and improve long-term outcomes.

In addition to cardiovascular disease, IBD has also been associated with other cardiac complications. One of the most relevant is atrial fibrillation, likely influenced by chronic inflammation and frequent hydroelectrolytic disturbances that can affect cardiac electrical conduction [123]. A nationwide Danish study involving 24,499 patients with IBD found a significantly increased incidence of atrial fibrillation during disease flares [IRR 2.63 (2.26–3.06)] and in patients with persistent inflammatory activity [IRR 2.06 (1.67–2.55)], but not during clinical remission periods [IRR 0.97 (0.88–1.08)] [124]. These findings support the recommendation of performing an electrocardiogram in any IBD patient presenting with tachycardia, especially during flare-ups, to allow early diagnosis and initiation of appropriate antiarrhythmic or anticoagulant therapy.

Besides, IBD has been linked to an increased risk of cerebrovascular thromboembolic events. Bernstein et al. reported a higher incidence of these events in patients with IBD, particularly in those with CD [IRR 1.26; 95% CI (1.05–1.66)] [125].

Another well-known cardiovascular complication in IBD is venous thromboembolism. Patients with IBD have a 3.5-fold increased risk of developing venous thromboembolism compared to the general population [126]. This risk is particularly elevated during periods of active disease, hospitalization, and following surgical procedures, reflecting the prothrombotic environment associated with systemic inflammation and immobilization [126]. The most common sites of involvement include the deep veins of the lower extremities and the pulmonary vasculature, followed by less frequent locations such as the portal and mesenteric veins [127]. Thromboprophylaxis during hospitalization and in the early post-discharge period has been shown to reduce the incidence of venous thromboembolism in this high-risk population [128]. However, the optimal duration of prophylaxis remains controversial.

Other more infrequent related complications are myocarditis and pericarditis, which may result from autoimmune responses or drug toxicity, particularly from 5-ASA derivatives [84,85]. Valvulopathies, such as aortic and mitral regurgitation, can result from chronic inflammation, as well as myxomatous degeneration [129]. Endocarditis, though rare, has been reported in patients with IBD, often associated with immunosuppression, central venous catheters, or underlying valvulopathies [130]. Finally, Takayasu arteritis, a large-vessel vasculitis, has been occasionally described in association with IBD, especially UC [131].

## 13. Metabolic Disorders

Dyslipidemia is a relevant metabolic complication in IBD due to its contribution to atherosclerosis and cardiovascular risk, as previously described [132,133]. Chronic systemic inflammation in IBD induces a pro-atherogenic lipid profile characterized by decreased levels of high-density lipoprotein cholesterol and increased low-density lipoprotein cholesterol. Paradoxically, patients with active IBD may present with reduced levels of total cholesterol and LDL-C compared to those in remission and the general population (probably related with intestinal malabsorption of cholesterol and fat), while HDL-C and triglyceride levels remain relatively unchanged [134]. Although IBD therapies can control systemic inflammation and can partially normalize lipid metabolism, corticosteroids and JAK inhibitors are associated with significant increases in serum lipid levels [135].

Impaired glucose metabolism and diabetes is another metabolic complication to consider in patients with IBD. Type 2 diabetes mellitus occurs more frequently in this population [126]. The principal contributing factor is the prolonged administration of systemic corticosteroids, which enhance hepatic gluconeogenesis, inhibit glucose uptake in peripheral tissues such as muscle and adipose tissue, and impair insulin signaling [136]. Additionally, chronic systemic inflammation inherent to IBD pathophysiology exacerbates insulin resistance through the action of proinflammatory cytokines (particularly TNF-α, IL-1, and IL-6) which disrupt the insulin signaling cascade [137].

Obesity is also a common comorbidity in patients with IBD, with its prevalence now comparable (or even higher) to the general population [138]. This trend reflects both the rising rates of obesity in the general population and improvements in IBD management that facilitate remission and promote anabolic metabolism. Additionally, therapies such as corticosteroids contribute to weight gain [94,95]. Obesity in IBD carries several clinical implications. Visceral adipose tissue acts as an endocrine, releasing proinflammatory cytokines such as TNF-α, IL-6, and leptin, which sustain systemic inflammation [139,140]. Moreover, obesity and dyslipidemia independently elevate cardiovascular risk. Their coexistence, along with hypertension and impaired glucose tolerance, constitutes metabolic syndrome, which exacerbates this high cardiovascular risk [141,142,143,144,145].

Altogether, these metabolic complications, along with chronic systemic inflammation, may contribute to the development of MASLD, which represents one of the most relevant hepatic complications in patients with IBD, as previously described [2,12].

## 14. Amyloidosis

Amyloidosis is a disorder characterized by the extracellular deposition of amyloid, an abnormal insoluble fibrous protein with a β-pleated sheet structure, in organs, leading to their dysfunction. Amyloidosis is classified based on etiology into types such as primary amyloidosis (AL) and secondary amyloidosis (AA) [146].

AL amyloidosis is typically idiopathic or associated with plasma cell dyscrasias like multiple myeloma, while AA amyloidosis occurs secondary to chronic inflammatory diseases, such as IBD [147]. Common clinical manifestations include nephrotic syndrome, congestive heart failure, and peripheral neuropathy. Although the incidence of amyloidosis in IBD is low (<3%), it can lead to severe organ impairment [148].

Subclinical amyloid deposits may be identified through Congo red staining of biopsy samples. Despite the importance of early detection, there is no consensus on the criteria for identifying patients with IBD at high risk of developing amyloidosis. Treatment of AA amyloidosis focuses on controlling the underlying inflammatory condition to reduce amyloid production [149].

## 15. Respiratory Manifestations in IBD

Respiratory involvement is a rare manifestation, appearing to be more common in UC than CD [150]. Although the incidence of thoracic manifestations is reported to be below 1%, recent studies suggest this may be underestimated. Pulmonary function test abnormalities have been observed in 40–60% of patients with IBD, and chest CT scans reveal anomalies in 22–88% of cases, often without clinical symptoms [151,152].

The pathophysiological mechanism is unclear, but the most widely accepted theory suggests a shared immunological basis. The tracheobronchial tree and the gastrointestinal tract originate from the same embryonic tissue and contain lymphoid structures with similar immune functions. This implies that the immune response in IBD could also target the lungs through mechanisms such as lymphocyte cross-sensitization, immune complex circulation, and/or cytokine release [153]. Additionally, certain IBD treatments (sulfasalazine, mesalazine, methotrexate, and anti-TNF agents) may cause pulmonary toxicity [154].

Although rare, upper airway involvement in IBD can be severe, presenting with dysphonia, stridor, due to inflammatory or pseudo-inflammatory lesions or glottic/subglottic stenosis. Prompt treatment with high-dose systemic corticosteroids is required, and in severe cases, temporary tracheostomy may be necessary [110,111].

Chronic tracheobronchitis and suppurative bronchitis are one of the most well-documented pulmonary manifestations of IBD. They typically present with persistent cough and mucopurulent sputum. Management includes controlling the underlying IBD and treatment with inhaled corticosteroids [155,156].

Bronchiectasis is the most common pulmonary manifestation in IBD [150,157]. It typically presents with chronic productive cough and mucopurulent sputum, often with infectious exacerbations. Diagnosis is established by CT scan [158]. Management includes respiratory physiotherapy, sputum culture to guide antibiotic therapy (considering atypical or resistant pathogens in the context of IBD-related immunosuppression), and, in selected cases, the use of low-dose cyclic macrolides may be beneficial [159].

Interstitial lung diseases (ILDs) are a heterogeneous group of conditions affecting the alveolar interstitium and pulmonary supporting structures. In the context of IBD, ILDs represent a relatively uncommon parenchymal manifestation but are frequently associated with drug-induced toxicity, particularly from sulfasalazine, mesalazine, and methotrexate [160]. Clinically, they may present with fever, dry cough, progressive dyspnea, and elevated acute-phase reactants, often mimicking antibiotic-resistant infectious pneumonia. Chest radiographs typically reveal patchy alveolar opacities, often peripheral, while CT imaging commonly shows multifocal consolidations. Management includes systemic corticosteroids and withdrawal of the causative agent when possible [161].

Other less common pulmonary manifestations of IBD include involvement of the small airways, such as various forms of bronchiolitis (including obliterative, chronic, and granulomatous types). These conditions are often subclinical or present with mild symptoms such as dry cough and exertional dyspnea. Diagnosis is typically made by CT scan. Most cases respond to systemic corticosteroids, although some may result in significant long-term sequelae [107,162].

Pleuritis is rare in IBD and may present as pleuritic chest pain or an exudative pleural effusion. Diagnosis is based on imaging and thoracentesis. Mesalazine could cause a mesalazine-induced lupus—characterized by arthralgias, serositis, and positive antinuclear antibodies—in this case, the drug should be discontinued. Standard treatment includes corticosteroids, and in cases of significant effusion, drainage may be required [119,163].

Pulmonary vasculitis is a very rare but severe complication of IBD, potentially leading to diffuse alveolar hemorrhage with symptoms such as dyspnea, hemoptysis, and respiratory failure. Management requires intensive treatment, including ventilatory support, corticosteroids, and other immunosuppressive agents. Anti-TNF may act as potential trigger; thus, their use should be reassessed in patients receiving this therapy [118,164].

## 16. Protein–Calorie Malnutrition

The prevalence of protein–calorie malnutrition in IBD varies widely across studies, with reported rates ranging from approximately 5% to 70%, depending on the diagnostic criteria used and whether patients were in remission or experiencing a disease flare [122,165].

Malnutrition in patients with IBD has a multifactorial etiology. One contributing factor is reduced oral food intake, often related to loss of appetite due to gastrointestinal symptoms such as nausea, vomiting, abdominal pain, and diarrhea, or as a result of drug-related side effects. In addition, caloric and protein intake may decline during hospitalizations [166]. Certain treatments used in IBD, such as immunosuppressants, may also contribute to anorexia [167,168].

Another contributing mechanism is nutrient malabsorption. Intestinal mucosal inflammation (particularly in CD with upper gastrointestinal tract involvement) leads to structural and functional alterations that impair nutrient absorption. Additionally, chronic inflammatory diarrhea results in continuous losses of fluids, electrolytes, and proteins, further exacerbating nutritional deficiencies [169]. Moreover, ileal CD can lead to fat malabsorption due to impaired bile salt reabsorption, resulting in deficiencies of fat-soluble vitamins. Extensive intestinal resections further increase the risk of short bowel syndrome and associated malabsorption [170].

Systemic inflammation also contributes to malnutrition by increasing basal energy expenditure and promoting a hypercatabolic state [126,127]. This condition also contributes to the loss of lean body mass, leading to sarcopenia [171].

There is no established gold standard for the diagnosis of protein–calorie malnutrition in IBD. In 2019, the Global Leadership Initiative on Malnutrition (GLIM) proposed diagnostic criteria applicable to all patient populations. According to GLIM, the diagnosis requires the presence of at least one phenotypic criterion (low BMI, unintentional weight loss, or reduced muscle mass) and one etiologic criterion (reduced food intake or impaired absorption, along with active inflammatory disease or significant metabolic burden) [172]. Protein–calorie malnutrition has significant clinical consequences in patients with IBD, negatively affecting disease progression and treatment outcomes [129,130,131,173,174,175,176].

Optimization of nutritional status using enteral or parenteral support, supplementation, correction of vitamin deficiencies, and appropriate dietary interventions is a key component of comprehensive IBD management [177].

## Figures and Tables

**Table 1:** Clinical screening and management recommendations for systemic consequences of inflammatory bowel disease.

Organ System	Main Systemic Complications	Clinical Advice for Screening/Risk Assessment	Key Management and Preventive Strategies
Hepatobiliary	Metabolic dysfunction–associated fatty liver disease (MAFLD) Drug-induced liver injury Gallstones	Perform liver function tests at baseline and periodically (every 6–12 months). Abdominal ultrasound in patients with metabolic risk factors, obesity, or prolonged corticosteroid use or if biliary pain.	Optimize metabolic control. Be aware and recognize hepatotoxic drugs. Manage overweight. Consider drug switch if persistent liver enzyme elevation.
Neurological	Peripheral neuropathy Demyelinating disease Neurodegeneration	Screen for neuropathic symptoms (paresthesia, weakness) in long-standing or malnourished IBD. Assess B12, folate, and thiamine levels annually.	Correct deficiencies. Avoid long-term neurotoxic drugs (e.g., metronidazole >3 weeks), Refer to neurology if demyelination suspected.
Psychological/Psychiatric	Fatigue Depression Anxiety Cognitive dysfunction	Annual screening using PHQ-9 and GAD-7; assess fatigue with IBD-Fatigue or FACIT-F scales. Investigate cognitive decline Investigate insomnia	Integrated care with psychology/psychiatry. Treat underlying inflammation. Correct anemia. Consider CBT or mindfulness therapy. Promote healthy sleeping habits.
Hematologic	Iron-deficiency anemia Anemia of chronic disease Cytopenias	Cell blood count and ferritin at diagnosis and every 6–12 months, or more frequently during flares.	Intravenous iron preferred in active disease. Treat inflammation Monitor drug toxicity (thiopurine)
Renal/Urological	Chronic kidney disease Nephrolithiasis Drug-induced nephritis	Check serum creatinine at least ever 6–12 months Evaluate for kidney stones in patients flank pain.	Ensure hydration Avoid/minimize nephrotoxic drugs (e.g., NSAIDs) Correct metabolic abnormalities.
Bone/Metabolic	Osteopenia Osteoporosis Fractures	DEXA scan at diagnosis if risk factors (steroids, malnutrition, postmenopausal); repeat every 2–3 years. Check vitamin D annually.	Calcium/vitamin D supplementation, reduce steroid exposure, encourage weight-bearing exercise, consider bisphosphonates if osteoporosis.
Musculoskeletal/Nutritional	Sarcopenia Protein–calorie malnutrition	Assess BMI and muscle mass (CT, ultrasound, or handgrip strength) annually. Evaluate dietary intake and weight trends.	Early nutritional intervention. Optimize protein intake. Recommend resistance exercise. Correct deficiencies. Treat inflammation.
Cardiovascular/Thromboembolic	Atherosclerotic disease Venous thromboembolism Arrhythmias	Assess cardiovascular risk factors annually (Blood pressure, smoking…). Consider ECG in active flares or palpitations. Provide VTE prophylaxis during hospitalization.	Lifestyle modification. Treat inflammation efficiently. Minimize steroids. Statins if indicated. Anticoagulate per guidelines.
Respiratory	Bronchiectasis Interstitial lung disease Drug-induced pneumonitis	Ask for chronic cough or dyspnea during follow-up. Consider chest imaging if symptomatic or exposed to suspect drugs (5-ASA, MTX).	Withdraw offending drug. Corticosteroids if immune-mediated. Pulmonary referral when persistent.
Dermatologic/Oral	Telogen effluvium Alopecia areata Oral ulcers	Evaluate hair loss and oral lesions in active or malnourished patients. Check ferritin, zinc, and B12 if alopecia or stomatitis.	Correct deficiencies. Control IBD activity. Topical therapy for oral lesions. Multidisciplinary approach if persistent.
Endocrine/Reproductive	Hypogonadism Infertility Sexual dysfunction	Assess fertility and sexual function routinely. Preconception counselling for all patients planning pregnancy.	Optimize disease control. Discontinue teratogenic drugs (MTX). Provide psychological and sexual health support.
Systemic/Amyloidosis	Secondary (AA) amyloidosis	Suspect in refractory inflammation with proteinuria or organ dysfunction. Perform serum amyloid A and biopsy if indicated.	Control inflammation efficiently. Nephrology referral.

ALT: Alanine aminotransferase; BMD, Bone mineral density; BP, Blood pressure; B12, Vitamin B12 (cobalamin); CBT, Cognitive behavioral therapy; CBC, Complete blood count; CT, Computed tomography; DEXA, Dual-energy X-ray absorptiometry; DILI, Drug-induced liver injury; ECG, Electrocardiogram; FACIT-F, Functional Assessment of Chronic Illness Therapy–Fatigue; GAD-7, Generalized Anxiety Disorder-7 scale; IGF-1, Insulin-like growth factor-1. LFT, Liver function test; MAFLD, Metabolic dysfunction–associated fatty liver disease; MRI, Magnetic resonance imaging; PHQ-9, Patient Health Questionnaire-9; ULN, Upper limit of normal; VTE, Venous thromboembolism.

**Table 2:** Drug-induced systemic complications of IBD therapies across organ systems.

Drug/Class	Main Organ Systems Affected	Typical Adverse Effects	Clinical Notes/Monitoring Advice
Aminosalicylates (5-ASA, mesalazine, sulfasalazine)	Hepatobiliary, Renal, Respiratory, Hematologic	Mild transaminase elevation, interstitial nephritis, lupus-like serositis, bone-marrow suppression (rare, more likely with sulfasalazine)	Monitor liver enzymes and creatinine every 6–12 months; discontinue if ALT > 3× ULN or rising creatinine; avoid rechallenge after confirmed toxicity.
Corticosteroids	Metabolic, Bone, Cardiovascular, Psychiatric, Ocular	Osteoporosis, weight gain, hyperglycaemia, hypertension, mood disturbance, cataracts, suicide ideation, depression and anxiety	Use lowest effective dose and shortest duration; consider bone protection; monitor BP, glucose, mood; taper gradually. Explore mental health impact.
Thiopurines (azathioprine, 6-MP)	Hepatobiliary, Hematologic, Dermatologic	Hepatotoxicity via 6-MMP accumulation, leukopenia, lymphoma risk, alopecia	Check TPMT/NUDT15 before start; monitor CBC and LFTs every 3 months; dose-adjust or discontinue if toxicity develops.
Methotrexate	Hepatobiliary, Pulmonary, Hematologic, Dermatologic	Transaminase elevation, hepatic fibrosis, pneumonitis, cytopenia, alopecia	Avoid in alcohol use or obesity; supplement folic acid; monitor CBC/LFTs; chest X-ray if respiratory symptoms.
Calcineurin inhibitors (cyclosporine, tacrolimus)	Renal, Neurological, Cardiovascular	Nephrotoxicity, tremor, hypertension, reversible posterior leukoencephalopathy	Monitor serum creatinine, BP, and drug levels; reduce dose or switch if neurotoxicity or nephrotoxicity occurs.
Anti-TNF agents (infliximab, adalimumab, certolizumab, golimumab)	Hepatobiliary, Neurological, Dermatologic, Respiratory	Drug-induced hepatitis, demyelinating disease, psoriasiform rash, interstitial lung disease.	Baseline hepatitis screen and LFTs; avoid in pre-existing demyelination; monitor for respiratory or dermatologic symptoms.
Anti-integrin therapy (vedolizumab)	Hepatobiliary	Mild transaminase elevation (rare)	Excellent hepatic safety overall; routine LFTs recommended.
Anti-IL-12/23 and IL-23 inhibitors (ustekinumab, risankizumab, mirikizumab)	Hepatobiliary, Dermatologic, Neurological	Mild ALT/AST elevation, rare hypersensitivity or rash Rare cases of posterior reversible encephalopathy syndrome (PRES)	Routine LFTs; discontinue only if confirmed DILI or if neurological adverse event suggestive of PRES.
JAK inhibitors (tofacitinib, filgotinib, upadacitinib)	Metabolic, Cardiovascular, Hepatobiliary, Hematologic	Hyperlipidaemia, transaminase rise, cytopenia, herpes zoster, increased MACE/VTE risk (especially with tofacitinib)	Baseline CBC, LFTs, and lipids; re-check at 4–8 weeks then every 3–6 months; avoid in patients with high CV risk.
S1P modulators (etrasimod, ozanimod)	Cardiovascular, Hepatic	Bradycardia, transaminase elevation (mild)	ECG before first dose; LFTs at baseline and periodically; avoid with severe hepatic impairment.
Antibiotics (metronidazole, ciprofloxacin)	Neurological, Musculoskeletal	Peripheral neuropathy, tendinopathy	Avoid long-term metronidazole (>3–4 weeks); stop if neuropathy develops; caution with fluoroquinolones in elderly.

*5-ASA*, 5-aminosalicylic acid; *ALT*, alanine aminotransferase; *AST*, aspartate aminotransferase; *BP*, blood pressure; *CBC*, complete blood count; *CV*, cardiovascular; *DILI*, drug-induced liver injury; *ECG*, electrocardiogram; *JAK*, Janus kinase; *LFT*, liver function test; *MAFLD*, metabolic dysfunction–associated fatty liver disease; *MACE*, major adverse cardiovascular events; *MTX*, methotrexate; *PRES*: posterior reversible encephalopathy syndrome; *S1P*, sphingosine-1-phosphate; *ULN*, upper limit of normal; *VTE*, venous thromboembolism.

## Data Availability

Not applicable. No new data were created or analyzed in this study. Data sharing is not applicable to this article.
